# New insights into the anticancer effects of *Polycladia crinita* aqueous extract and its selenium nanoformulation against the solid Ehrlich carcinoma model in mice via VEGF, notch 1, NF-кB, cyclin D1, and caspase 3 signaling pathway

**DOI:** 10.3389/fphar.2024.1345516

**Published:** 2024-02-16

**Authors:** Badriyah S. Alotaibi, Thanaa A. El-Masry, Hend Selim, Maisra M. El-Bouseary, Mostafa M. El-Sheekh, Mofida E. M. Makhlof, Maysa M. F. El-Nagar

**Affiliations:** ^1^ Department of Pharmaceutical Sciences, College of Pharmacy, Princess Nourah bint Abdulrahman University, Riyadh, Saudi Arabia; ^2^ Department of Pharmacology and Toxicology, Faculty of Pharmacy, Tanta University, Tanta, Egypt; ^3^ Department of Biochemistry, Faculty of Pharmacy, Tanta University, Tanta, Egypt; ^4^ Department of Microbiology and Immunology, Faculty of Pharmacy, Tanta University, Tanta, Egypt; ^5^ Botany Department, Faculty of Science, Tanta University, Tanta, Egypt; ^6^ Botany and Microbiology Department, Faculty of Science, Damanhour University, Damanhour, Egypt

**Keywords:** anticancer, brown algae, macroalgae, selenium nanoparticles, solid Ehrlich carcinoma

## Abstract

**Background:** Phaeophyceae species are enticing interest among researchers working in the nanotechnology discipline, because of their diverse biological activities such as anti-inflammatory, antioxidant, anti-microbial, and anti-tumor. In the present study, the anti-cancer properties of *Polycladia crinita* extract and green synthesized *Polycladia crinita* selenium nanoparticles (PCSeNPs) against breast cancer cell line (MDA-MB-231) and solid Ehrlich carcinoma (SEC) were investigated.

**Methods:** Gas chromatography–mass spectroscopy examinations of *Polycladia crinita* were determined and various analytical procedures, such as SEM, TEM, EDX, and XRD, were employed to characterize the biosynthesized PCSeNPs. *In vitro,* the anticancer activity of free *Polycladia crinita* and PCSeNPs was evaluated using the viability assay against MDA-MB-231, and also cell cycle analysis by flow cytometry was determined. Furthermore, to study the possible mechanisms behind the *in vivo* anti-tumor action, mice bearing SEC were randomly allocated into six equal groups (n = 6). Group 1: Tumor control group, group 2: free SeNPs, group 3: 25 mg/kg *Polycladia crinita*, group 4: 50 mg/kg *Polycladia crinita*, group 5: 25 mg/kg PCSeNPs, group 6: 50 mg/kg PCSeNPs.

**Results:** Gas chromatography–mass spectroscopy examinations of *Polycladia crinita* extract exposed the presence of many bioactive compounds, such as 4-Octadecenoic acid-methyl ester, Tetradecanoic acid, and n-Hexadecenoic acid. These compounds together with other compounds found, might work in concert to encourage the development of anti-tumor activities. *Polycladia crinita* extract and PCSeNPs were shown to inhibit cancer cell viability and early cell cycle arrest. Concentrations of 50 mg/kg of PCSeNPs showed suppression of COX-2, NF-кB, VEGF, ki-67, Notch 1, and Bcl-2 protein levels. Otherwise, showed amplification of the caspase 3, BAX, and P53 protein levels. Moreover, gene expression of caspase 3, caspase 9, Notch 1, cyclin D1, NF-кB, IL-6, and VEGF was significantly more effective with PCSeNPs than similar doses of free extract.

**Conclusion:** The PCSeNPs mediated their promising anti-cancerous action by enhancing apoptosis and mitigating inflammation, which manifested in promoting the total survival rate and the tumor volume decrease.

## Graphical Abstract

**Figure:**
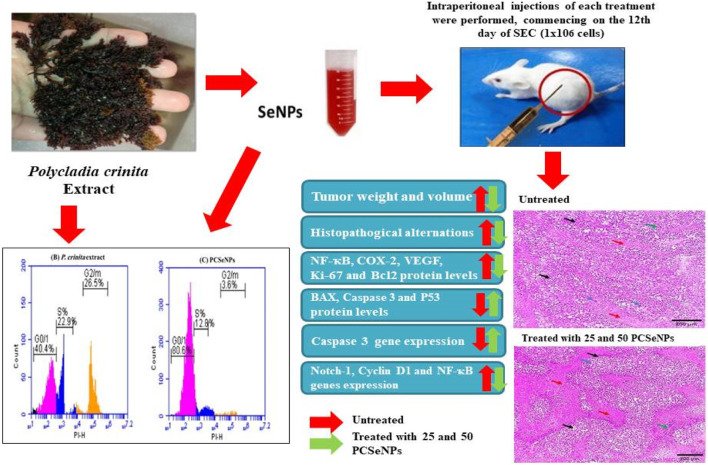
SeNPs, selenium nanoparticles; *P. crinita*, *Polycladia crinita*; and PCSeNPs, *Polycladia crinita* selenium nanoparticles.

## 1 Introduction

Cancer develops when the homeostatic equilibrium between cell growth and death is disturbed (Amin et al., 2019). This contributes to the dysregulation of the normal cellular system for cell division, cell differentiation, and cell death, and ultimately to a group of cells that can metastasize to other vital organs, causing substantial morbidity (El Bakary et al., 2020). Its treatment is still a matter of challenge despite the advanced understanding of the molecular basis involved. Chemotherapy and other standard cancer therapies can be effective, but they also tend to be toxic and have a deleterious effect on the health of patients. Therefore, finding alternative therapies that can eradicate cancer cells, while also having the benefit of fewer side effects that can enhance patient health needs to be urgently addressed (Moncevičiute-Eringiene, 2005; Wang J. et al., 2012; Badr El-din et al., 2020; ElSaied et al., 2021).

Natural products and their derivatives are valuable resources for the discovery of novel small-molecule cancer therapy agents (Li and Weng, 2017). Seaweed’s natural products can be categorized as red, brown, or green algae. Within each category, there are many bioactive chemicals with various characteristics that can be utilized for biotechnological purposes (Hirmo et al., 1995). Because seaweed has many bioactive chemicals, they are used in biomedical applications such as antibacterial (Adhikari et al., 2006), antiviral (Bhosale et al., 2002), anti-inflammatory, anticoagulant, and antithrombotic (Soo-Jin et al., 2005; Khotimchenko et al., 2020). Furthermore, Brown algae have antitumoral action against some cancer cell proliferation without adversely affecting healthy cells, as is the case with existing antitumoral therapies (Pádua et al., 2015; Almurshedi et al., 2023).

*Polycladia crinita* is a natural brown seaweed that has proven to show anti-inflammatory, antioxidant, and anticancer activity (Asokapandian et al., 2021). *Polycladia crinita* extract indicated the presence of several bioactive components. Fatty acids (FA) were the primary classification for these substances (Almurshedi et al., 2023). Fatty acids reduce plasma triglyceride levels and reduce the risk of cardiovascular diseases, among other positive impacts on human health (Casula et al., 2013). They also have neuroprotective properties (Wall et al., 2010), and anti-inflammatory properties (Kyle et al., 1999; Harris et al., 2013). Furthermore, the consumption of fatty acids may inhibit the development of malignancies by causing apoptosis and inhibiting angiogenesis (Serini et al., 2009; Bi et al., 2019). They not only have a strong safety record but also increase the efficiency of chemotherapeutic drugs and mitigate the side effects of chemotherapy or cancer (Hardman, 2002; Leaver et al., 2002; Spencer et al., 2009). In addition, Fucosterol which was identified in *Polycladia Crinita* has shown anti-cancer properties (Siddiqui et al., 2011).

Nowadays, bioactive molecules, including natural nutritional elements and algal components allied with nanoparticles, are being investigated as potential replacements for conventional anticancer agents (Miller et al., 2019; Bukowski et al., 2020). For example, For example,; in HepG2 carcinoma cells, *Polycladia myrica* halts cellular proliferation, in addition, it showed a promising role alone or with radiation in defeating breast cancer cell progression in Ehrlich carcinoma-induced in mice (Abo-Neima et al., 2023).

The fundamental unit of nanotechnology, known as a nanoparticle (NP), has at least one dimension between 1 and 100 nm. These substances are used in a variety of sectors including agricultural activities, sewage treatment, electronics, heavy metals adsorption, skincare industry, and medical practices, due to their distinctive characteristics (Palani et al., 2021; Fouda et al., 2022). The fabrication of nanoparticles and nano-colloids has been achieved via biological, chemical, and physical processes (Datta et al., 2022).

The biological manufacturing of nanoparticles utilizing extracts from plants and microorganisms, such as bacteria, fungi, and algae, is particularly intriguing since it protects from pressure, high temperatures, and potentially toxic reagents. Due to the capping effect of the algal extract, green-synthesized nanoparticles have a high degree of stability (Chen et al., 2020; Heinemann et al., 2021). Additionally, the method is economical, appropriate for biological use, and ecologically beneficial (Gour and Jain, 2019; Yazdanian et al., 2022). Algae are particularly appealing for green synthesis among the different biological processes employed for manufacturing nanoparticles because of their versatility and usability. Furthermore, a lot of chemical constituents identified in algal extracts can serve various purposes, such as reducing and/or stabilizing agents for the biosynthesis of nanoparticles (Akintelu et al., 2020; Hano and Abbasi, 2021; Kaur and Thombre, 2021).

Selenium has attracted increasing attention in recent years due to its critical role in maintaining good health (Rayman, 2020). Selenium is essential for the metabolism of thyroid hormones and other vital metabolic activities, as well as for healthy immune system functioning. By getting integrated into antioxidant enzymes, it additionally stops cell destruction caused by oxidative stress. Numerous dangerous disorders, including malignancy, cardiovascular, and inflammation-related conditions, have been associated with selenium inadequacy (Misra et al., 2015). Nevertheless, prolonged Se supplements or greater doses may be harmful (Misra et al., 2015; Rayman et al., 2018). SeNPs have come under the spotlight recently. Reports about their synthesis and use are ongoing (El-Ramady et al., 2016). Comparing both inorganic and organic forms of selenium, SeNPs' reported toxicity was lesser (Bhattacharjee et al., 2019).

The most common cancer in women’s world widely is breast cancer. It hits about 24.5% of all women around the world (Globocan, 2008). Breast cancer counts as the second cause of death among women (El-Ashmawy et al., 2022). Above one million new cases of breast cancer are identified worldwide yearly, according to the World Health Organization, establishing significant financial, emotional, and health-related consequences (Gu et al., 2018; El-Sherbiny et al., 2021).

In the context of breast cancer development, Notch has been identified as one of the leading drivers of cellular proliferation and cancer progression (Kontomanolis et al., 2018). It has been reported for its ability to trigger cyclin expressions, hence increasing cellular division, as well as enhancing angiogenesis and suppressing apoptosis. There is entanglement between Notch and inflammatory mediator, NF-кB, which, in turn, activates the expression of other inflammatory mediators and vascular endothelial growth factor expression (El-Sherbiny et al., 2021; Jiang et al., 2022).

Based on all the above-mentioned evidence, there is a great interest in recognizing the potential antitumor effect of *Polycladia crinita*. Solid Ehrlich carcinoma (SEC) is one of the models used for breast cancer investigation due to its similarity to undifferentiated solid mass with a rapid growth rate (Amin et al., 2019). Therefore, this study is the first of its kind that aimed to uncover whether the *Polycladia crinita* extracts have potential anticarcinogenic activity and the possible underlying mechanisms using SEC as a tumor model.

## 2 Materials and methods

### 2.1 Materials

The chemicals were all of analytical purity and were acquired from Sigma Aldrich. For the manufacture of SeNPs, sodium selenite (Na_2_SeO_4_) was employed as a precursor.

### 2.2 *Polycladia crinita* biomass collection

*Polycladia crinita*, a brown algae, was collected off the shore of the Gulf of Suez in Egypt. An investigator from NIOF Egypt named Dr. Fekry Mourad identified the algae. Aleem’s methods (Aldubayan et al., 2019; Vikneshan et al., 2020) were followed to identify every sample, and the data indicated above had been confirmed on the Algae Base website (Gardouh et al., 2020).

### 2.3 *Polycladia crinita* aqueous extract preparation

The biomass of *Polycladia crinita* was collected, rinsed with double-distilled water to get rid of any adhered particles and sludge, and then repeatedly washed with tap water to get rid of salt. After being washed and oven-dried for 15 min at 60°C, the samples were electric mixer-ground into fine dust. About 10 g of the algal powder was added to 100 mL of distilled water, well mixed, and heated to 60°C for 2 hours with a magnetic stirrer. After centrifuging the mixture for 10 min at 1,500 rpm (Fouda et al., 2022), the liquid that was produced was collected.

### 2.4 GC-MS analysis of *Polycladia crinita* aqueous extract

*Polycladia crinita* aqueous extract was subjected to Thermo Scientific TRACE 1310 Ga Chromatograph attached with ISQ LT single quadrupole Mass Spectrometer. GC-MS analysis was performed by injecting 1 μL of sample into the column DB5-MS, 30m; 0.25 mm ID (J&W Scientific) with helium as a carrier gas (Flow rate 1 mL/min). The program was as follows: 40°C (3 min) - 280°C (5 min) at 5°C/min, −290°C (1 min) at 7.5°C/min, detector temperature, 300°C, injector temperature: 200°C, and ionization voltage:70eV. Components were identified by comparing their mass spectra with those in the database of Wiley and Nist mass spectral database.

### 2.5 *Polycladia crinita* SeNPs (PCSeNPs) biosynthesis

The recovered liquid was used to reduce and stabilize SeNPs by combining the *Polycladia crinita* extract with 1 mM Na_2_Seo_4_ at a 1:9 ratio. The finished product’s color changed from light brown to an intense brown after being incubated in a dark, shaking environment for the whole night. This shift indicated the synthesis of PCSeNPs (Didžiapetrienė et al., 2020a). The resulting PCSeNPs were then produced by centrifugation at 10,000 rpm for 30 min, followed by a water wash, pure alcohol processing, heating to 50°C, storage in a sealed box, and use in any future assessments or study (Aleem, 1978).

### 2.6 Characterization of the synthesized *Polycladia crinita* SeNPs (PCSeNPs)

Using a UV-Vis spectrophotometer, the highest surface plasmon resonance (SPR) of the synthesized PCSeNPs was determined. Using a UV-Vis spectrophotometer (Thermo Scientific Evolution TM 300, Thermo Fisher Scientific, United States of America) to measure the absorbance spectra in the 200–800 nm region, PCSeNPs were found (Mandal et al., 2022). To analyze the sizes and forms of produced PCSeNPs, Transmission Electron Microscopy (TEM; JEM 2100, JEOL, Ltd., Tokyo, Japan) was employed (Gheda et al., 2021).

Scanning electron microscopy combined with energy-dispersive X-ray (SEM-EDX) (JSM 6490 LV, JEOL, Ltd., Tokyo, Japan) was used to analyze the PCSeNPs specimen’s elemental components (Wang et al., 2015). A 2θ degree range of 0°–80° was used to examine the X-ray diffraction (XRD) data using the XRD 6000 detector (Shimadzu Corp., Kyoto, Japan). The x-ray source was 2.2 KW Cu anode radiation, where k = 1.54184, and the operational conditions were voltage at 30 kV and current at 10 mA (Zhong et al., 2016).

### 2.7 *In vitro* study

#### 2.7.1 Anticancer activity of *P. crinita* extract and PCSeNPs against breast cancer cell line (MDA-MB-231) by using the viability assay

The Breast cancer cell line (MDA-MB-231) cell line used in this study was obtained from the National Cancer Institute, Cairo, Egypt. Using corning 96-well tissue culture plates the tumor cells were suspended in the medium at a concentration of 5 × 10^4^ cells/well and incubated in 37°C humid atmosphere with 5% carbon dioxide. Subsequent 48 h of exponential growth, the cells were incubated with *Polycladia crinita* extract and PCSeNPs at concentrations of 0, 3.13, 6.25, 12.5, 25 and 50 (µg ml^−1^, 48 h). After that, 10 µL of the 12 mM MTT stock solution (Vybrant^®^ MTT Cell Proliferation Assay Kit, (V-13154)) was added to each well and incubated at 37°C for 4 h. Then, 50 µL of DMSO was added to each well, mixed thoroughly with the pipette, and incubated at 37°C for 10 min. The absorbance was read (540nm; microplate reader, ELx 800, Bio-Tek Instruments Inc., United States of America) (Mosmann, 1983). The inhibition value was determined using the following equation:

The rate of inhibition (%)=(A/B) × 100, where A represents the treated cell’s optical density and B the untreated cells. Furthermore, IC5_0_ was computed by using GraphPad Prism software (San Diego, CA, United States).

#### 2.7.2 Cell-cycle analysis

The breast cancer cell line (MDA-MB-231) cell-cycle distribution after treatment with *P. crinita* extract and PCSeNPs was analyzed using flow cytometric analysis on the IC_50_ concentrations detected by MTT assay. Following *P. crinita* extract and PCSeNPs stimulation of the cells, the culture media was carefully withdrawn, PBS was added, gently shaken, and then PBS was removed. One milliliter of trypsin was added, given a good shake, and allowed to digest in the incubator. Following digestion, the cells were taken out of the incubator and put in a 3 mL medium with serum to finish off the trypsin digestion. Using a pipette, the cells were resuspended and transferred to the centrifuge tube (Xie et al., 2017). Then 3 mL PBS resuspension cells were added, the supernatant was removed by spinning at 1,000 rpm at room temperature for 5 min, and the supernatant was removed by centrifugation at normal temperature for 5 minutes at 1,000 rpm. After adding 75% alcohol to revive the cells, they were kept overnight at 4°C in a refrigerator. Centrifuged at 1,000 rpm for 5 minutes at room temperature, then collected the supernatant, added PBS to wash three times, added PI staining solution, stained for 30 minutes at 37°C, and used flow cytometry to determine the cell cycle (BD Accuri™ C6 Plus Flow Cytometer) (Yakonov et al., 2021). Using Accuri™ C6 software (BD Biosciences) the percentage of cells in each cell cycle phase was determined.

### 2.8 *In vivo* study

#### 2.8.1 Animals

Seventy female Swiss albino mice (weighing between 18 and 22 g and approximately 6–8 weeks of age) were obtained from the National Research Center (NRC) in Cairo, Egypt. Animals were kept for acclimatization (for 1 week) and supplied with filtered water and a standard pelleted diet (IBEX feed for research animals, Ibex International Co., Ltd., Cairo, Egypt). Animal handling was performed according to the guidelines for the care and use of laboratory animals approved by the research ethics committee of the Faculty of Pharmacy, Tanta University (TP/RE/11/22p-0064). To induce a tumor, the mice had been injected subcutaneously in the right flank with 1 × 10^6^ live EAC cells (1 × 10^6^ cells). A detectable solid tumor manifested after approximately 12 days (Shaheen and Fouda, 2018).

#### 2.8.2 Experimental design

Mice bearing SEC were randomly allocated into six equal groups (n = 6). The animal care providers, the lab technicians, and the histopathology experts were blinded regarding which mouse received treatment and the nature of the treatment provided to the animals to avoid any biases. Group 1: Tumor control group, where mice received the vehicle, saline, group 2: mice were given free SeNPs, group 3: mice were given 25 mg/kg *Polycladia crinita*, group 4: mice were given 50 mg/kg *Polycladia crinita*, group 5: mice were given 25 mg/kg *Polycladia crinita* loaded on SeNPs, group 6: mice were given 50 mg/kg *Polycladia crinita* loaded on SeNPs. Doses were chosen based on a pilot study in addition to earlier studies on related species (Zhong et al., 2016; Siraj et al., 2021). Six intraperitoneal injections of each treatment were performed, commencing on the 12th day of SEC induction and continuing until the 28th day. Finally, all mice were euthanized, and the euthanasia was performed by cervical dislocation (CD) according to the American Veterinary Medical Association (AVMA) Guidelines for the Euthanasia of Animals (2020 Edition). Tumors were extracted and divided into parts. One portion was preserved in 10% formalin for histopathological analysis, and the remaining portion was stored in a freezer at −80°C for additional biochemical investigations.

### 2.9 Measurement of survival rate

Throughout the study, the mice’s survival rate was tracked and reported using the Kaplan-Meier survival curve, which was calculated using the following formula: survival rate = number of surviving mice in a group/total number of mice during the experiment (28 days) after 1, 2, 3, and 4 weeks.

### 2.10 Tumor weight and volume

Tumor tissue was carefully removed after euthanasia and weighed with a precise electronic balance (ADAM^®^, UK). The tumor mass was measured using a Vernier digital caliper (Anyi Instrument Co., China) starting on the 12th day, and then day after day till the last day of the experiment. Then the tumor volume was calculated according to the formula: tumor volume (mm^3^) = 0.52 AB^2^, where A and B are the lengths of the minor and major axis lengths, respectively (Didžiapetrienė et al., 2020b).

### 2.11 Determination of VEGF, NF-кB, Notch-1, and cyclin D1 content

Enzyme-linked immunosorbent assay (ELISA) kits were purchased from Abcam and Glory Science Co. to determine the levels of VEGF and NF-кB in tumor tissues. The instructions were followed exactly as directed by the manufacturer. Also, Notch-1 and Cyclin D1 were evaluated via ELISA kits obtained from RayBiotech according to manufacturer protocol.

### 2.12 Determination of caspase 3, caspase 9, notch 1, cyclin D1, NF-кB, IL-6 and VEGF genes expression

The relative gene expression of caspase 3, caspase 9, Notch 1, Cyclin D1, NF-кB, IL-6, and VEGF was evaluated by qRT-PCR employing GAPDH as a housekeeping gene. The sequences of primers are listed in Table 1. The extraction of total RNA was achieved using TRIzol reagent (15,596,026) (Life Technologies, United States).

**TABLE 1:** Primers sequence.

Gene	Primers sequence (5′–3′)	References
Caspase 3	F: GGA​GTC​TGA​CTG​GAA​AGC​CGA​A	Casp3 Mouse qPCR Primer Pair (NM_009810), MP201794, OriGene Technologies, Inc
	R: CTT​CTG​GCA​AGC​CAT​CTC​CTC​A	
Caspase 9	F: GCT​GTG​TCA​AGT​TTG​CCT​ACC​C	Casp9 Mouse qPCR Primer Pair (NM_015733), MP201800, OriGene Technologies, Inc
	R: CCA​GAA​TGC​CAT​CCA​AGG​TCT​C	
Notch 1^a^	F: TCA​ATG​CCG​TGG​ATG​ACC​TA	Notch1 Mouse qPCR Primer Pair (NM_008714), MP209021, OriGene Technologies, Inc
	R: CCT​TGT​TGG​CTC​CGT​TCT​TC	
Cyclin D1	F: GCA​GAA​GGA​GAT​TGT​GCC​ATC​C	Ccnd1 Mouse qPCR Primer Pair (NM_007631), MP203092, OriGene Technologies, Inc
	R: AGG​AAG​CGG​TCC​AGG​TAG​TTC​A	
NF-кB^a^	F: AGC​GGG​AAC​TGA​GTG​AGA​TGA	Zhang et al. (2022)
	R: GCA​CCC​AGG​TTG​TAT​CGG​G	
IL-6^a^	F: TAC​CAC​TTC​ACA​AGT​CGG​AGG​C	IL-6 Mouse qPCR Primer Pair (NM_031168), MP206798, OriGene Technologies, Inc
	R: CTG​CAA​GTG​CAT​CAT​CGT​TGT​TC	
VEGF^a^	F: GTC ACT ATG CAG ATC ATG CGG A	Li et al. (2015)
	R: GTC ACT ATG CAG ATC ATG CGGA	
GAPDH^a^	F: CAT​CAC​TGC​CAC​CCA​GAA​GAC​TG	Gapdh Mouse qPCR Primer Pair (NM_008084), MP205604 OriGene Technologies, Inc
	R: ATG​CCA​GTG​AGC​TTC​CCG​TTC​AG	

^a^ Notch 1: Neurogenic locus notch homolog protein 1; NF-кB: Nuclear factor kappa B; IL-6: Interleukin 6; VEGF: vascular endothelial growth factor; GAPDH: Glyceraldehyde 3-phosphate dehydrogenase.

The reverse transcription process was performed by QuantiTects Reverse transcription kit (Qiagen, United States). The reaction mixtures consisted of complementary DNA amplicons, primers, and Syber green master mix (Maxima SYBR Green/qPCR Master Mix, Thermo Fisher Scientific, United States). The Livak method was employed to compute the fold change in the gene expression in comparison to the control group (calibrator) (Livak and Schmittgen, 2001).

### 2.13 Histopathological examination

Sections of tumor tissue were arranged (3–5 µm thick) and stained with hematoxylin and eosin (H&E). The characteristic histopathological features were examined under light microscopy. Necrosis was graded using a scale of 1–4 points as follows: 0 indicates absence, 1 (0%–20%), 2 moderate (21%–50%), 3 marked (51%–80%), and 4 indicates a diffuse pattern, which indicates the most severe degree (81%–100%) (Co et al., 2019).

### 2.14 Immunohistochemical examination

The immunohistochemical staining procedure was used according to (Saber et al., 2019), for antigen retrieval, dewaxed sections were put in a citric acid buffer solution of 0.05 M, pH 6.8. The sections were then treated with 0.3% H_2_O_2_ and protein block. After that, incubated with Bax antibody (Santa Cruz, Cat: sc-7480, 1:100 dilution), caspase-3 polyclonal antibodies (Invitrogen, Cat: PA5-77887, dilution 1/100), p53 antibody (Santa Cruz, Cat: sc-126, 1:100 dilution), Bcl-2 antibody (Santa Cruz, Cat: sc-7382, 1:100 dilution), COX-2 antibody (Santa Cruz, Cat: sc-19999, 1:100 dilution), and Ki-67 (Santa Cruz, Cat: sc-23900, 1:100 dilution). Then for 30 min at 37°C with a secondary antibody linked to horseradish peroxidase. Following each process, phosphate buffer saline was applied to the slides three times. Sections were exposed for 3 min to the 3,3′-diaminobenzidine tetrahydrochloride reagent. Finally, slides were counterstained with Mayer’s hematoxylin, washed with distilled water, and mounted with DPX.

Using a digital camera attached to a microscope (Olympus CX21, Japan), slides were inspected under a microscope, and digital micrographs were taken. By using ImageJ software; version 1.54 D, Java 1.8.0_354, Positive expressions of staining intensity were measured and presented as a percentage of positive area per 6 high power fields.

### 2.15 Statistics

Employing the SPSS 25 software, the data were analyzed by ANOVA followed by LSD *post hoc* test. The data are expressed as the means ± SD and the statistical significance was set at *p* < 0.05.

## 3 Results

### 3.1 GC-MS analysis of *Polycladia crinita* extract

**FIGURE 1:**
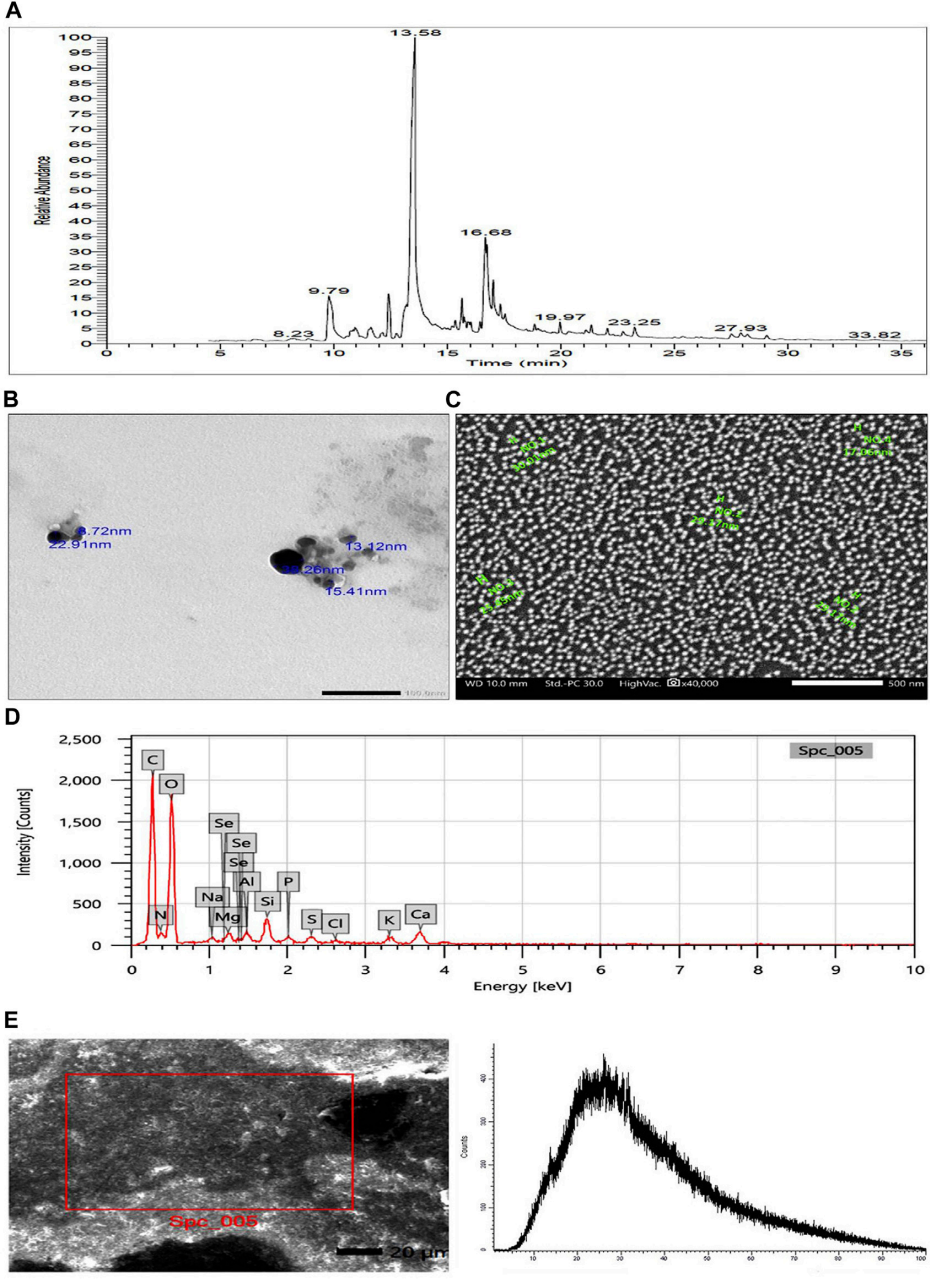
**(A)** GC-MS chromatogram of *Polycladia crinita* aqueous extract. **(B)** TEM imaging of *Polycladia crinita* mediated selenium nanoparticles (PCSeNPs), showing different particle sizes and morphology at scale bar 100 nm. **(C)** SEM imaging of *Polycladia crinita* mediated selenium nanoparticles (PCSeNPs), showing different particle sizes and morphology at scale bar 500 nm. **(D)** The EDX analysis of *Polycladia crinita* mediated selenium nanoparticles (PCSeNPs). **(E)** *Polycladia crinita*-mediated selenium nanoparticles (PCSeNPs) produced some background noise in the XRD pattern.

The GC-MS chromatogram (Figure 1A) demonstrated different bioactive compounds, counting 4-octadecenoic acid, methyl ester (66.13%), Tetradecanoic acid (60.57%), n-Hexadecenoic acid (45.66%), Octadecenoic acid (45.47%), Hexadecanoic acid, methyl ester (38.5%), Trans-13-Octadecenoic Acid (35.11%), Cis-13-Octadecenoic acid (15.88%) and Doconexent (14.68%). In addition, many other components were found and their bioactive properties are listed in Table 2.

**TABLE 2:** GC-MS analysis of Polycladia crinita extract.

RT^a^	Compound name	PA^a^ %	MF^a^	MW^a^	Biological activity
9.79	Tetradecanoic acid	8.56	C_14_H_28_O_2_	228	Fatty acids, antioxidant activity, and antimicrobial
10.87	Phytol	0.69	C_20_H_40_O	296	Precursor for the manufacture of synthetic forms of vitamin E and vitamin K1
10.95	2-Pentadecanone, 6,10,14 trimethyl	1.43	C_18_H_36_O	268	Essential oil, antioxidant activity, antimicrobial and anti-inflammation
11.64	Cyclohexanecarboxylic acid, hexadecyl ester	2.18	C_24_H_46_O_2_	366	Antimicrobial
12.16	1-Hexadecanol, 2-methyl	0.72	C_17_H_36_O_6_	256	Fatty acids, antioxidant activity, and antimicrobial
12.42	Hexadecanoic acid, methyl ester	5.93	C_17_H_34_O_2_	270	Fatty acids, antioxidant activity, and antimicrobial
12.77	R-Limonene	0.62	C_10_H_16_O_3_	184	Antioxidant activity
13.09	Trans-13-Octadecenoic acid	2.06	C_18_H_34_O_2_	282	Fatty acids, antioxidant activity, and antimicrobial
13.57	n-Hexadecenoic acid	43.80	C_16_H_32_O_2_	256	
15.36	9-Hexadecenoic acid	0.97	C_16_H_30_O_2_	254	
15.65	4-Octadecenoic acid, methyl ester	2.98	C_19_H_36_O_2_	296	
16.04	Octadec-9-enoic acid	0.61	C_18_H_34_O_2_	282	
16.68	Cis-13-Octadecenoic acid	13.52	C_18_H_34_O_2_	282	
17.03	Octadecenoic acid	2.52	C_18_H_36_O_2_	284	
17.34	Oleic Acid	1.13	C_18_H_36_O_2_	282	Fatty acids, antioxidant activity, lower cholesterol, antimicrobial, and anticancer
18.85	Cis-5,8,11,14,17-Eicosapentaenoic acid	0.67	C_20_H_30_O_2_	302	Synthesized from the essential fatty acid alpha-linolenic acid, regular heartbeat, and pumping function, and lessen blood clots
19.97	Fenretinide	1.01	C_26_H_33_NO_2_	391	Retinoid derivative inhibits the growth of several human cancer cell lines
22.05	Falcarinol	0.66	C_17_H_24_O	244	Antimicrobial
23.25	Doconexent	1.07	C_22_H_32_O_2_	328	Omega-3 fatty acid, Reduces triglycerides, is an anti-inflammatory, vasodilating, anti-arrhythmic, antioxidant, and inhibits the development and division of cancer cells as well as their ability to create new blood vessels
27.93	4,7,10,13,16,19-Docosahexaenoic acid, methyl ester	0.52	C_23_H_34_O_2_	342	Fatty acids, antioxidant activity

^a^ RT: retention time; PA: peak area; MF: molecular formula; MW: Molecular weight. The biological activities of different compounds were driven from the PubChem database website https://pubchem.ncbi.nlm.nih.gov/

### 3.2 Characterization of *Polycladia crinita* selenium nanoparticles (PCSeNPs)

The solution was light brown at the start of the synthesis procedure and did not alter in color. When the liquid turned dark brown after 48 h of reaction, it was visually confirmed that the sodium selenite had been reduced.

### 3.3 Transmission electron microscopy (TEM)

The smooth, spherical-shaped selenium nanoparticles produced by *P. crinita* extract’s reducing power for sodium selenite are visible in the TEM picture (Figure 1B). The selenium nanoparticles have a packed backdrop and range in size from 8.72 to 38.26 nm on average.

### 3.4 Scanning electron microscopy (SEM)

The formation of selenium nanostructures was further illustrated by the SEM picture showing the high-density selenium nanoparticles produced by processing *P. crinita* extract (Figure 1C). The selenium nanoparticles appear to range in average mean size from 17.06 to 30.01 nm.

### 3.5 Energy-dispersive X-ray analysis

The components and atomic content of PCSeNPs were determined using the energy-dispersive X-ray spectrometer (Figure 1D). Upon analyzing the EDX spectrum, it was determined that the mass percent of selenium was 0.26 ± 0.07, while the mass percents of oxygen and carbon were found to be the highest at 46.31 ± 0.4 and 39.96 ± 0.02, respectively. This confirms the formation of selenium nanoparticles along with the presence of numerous other elements derived from the components of the *P. crinita* extract, such as Mg, S, K, P, Al, Si, and Ca.

### 3.6 X-ray diffraction analysis

The crystallinity of phyco-synthesized SeNPs was assessed using the XRD pattern. Noisy backgrounds were shown by the data shown in (Figure 1E).

### 3.7 *In vitro* anticancer activity of free *Polycladia crinita* extract and PCSeNPs assessed using the viability assay against breast cancer cell line (MDA-MB-231)

Table 3 and Table 4 presented the inhibitory effect of free *P. crinita* extract and PCSeNPs on breast cancer cell line (MDA-MB-231) with IC_50_ = 45.33 ± 3.37 μg/mL and 20.62 ± 1.41 μg/mL, respectively. free *P. crinita* extract was used at concentrations of 0–50 μg/mL, the lowermost concentration (3.13 μg/mL) recorded the highest viability of 91.69% ± 0.82% and lower inhibition effect of 8.31% ± 0.12%, however with the highest inhibition of 52.9% ± 0.93% recorded at the highest concentration of free *P. crinita* extract (50 μg/mL) (Table 3).

**TABLE 3:** Activity of free *Polycladia crinita* extract against breast cancer cell line (MDA-MB-231, incubation for 48 h) with IC_50_ = 45.33 ± 3.37 μg/mL.

Free *P. crinita* extract concentration (µg/mL)	Viability (%)	Inhibition (%)
0	100	0
3.13	91.69 ± 0.82	8.31 ± 0.12
6.25	77.26 ± 0.77	22.74 ± 0.24
12.5	65.12 ± 0.55	34.89 ± 0.42
25	60.36 ± 0.75	39.63 ± 0.52
50	47.11 ± 0.32	52.9 ± 0.93

Data are expressed as mean ± SD, n = 3.

**TABLE 4:** Activity of PCSeNPs against breast cancer cell line (MDA-MB-231, incubation for 48 h) with IC_50_ = 20.62 ± 1.41 μg/mL.

PCSeNPs concentration (µg/ml	Viability (%)	Inhibition (%)
0	100	0
3.13	86.50±0.62	13.5±0.22
6.25	73.67±0.31	26.39±0.15
12.5	60.2±0.25	39.8±0.35
25	46.54±0.39	53.45±0.38
50	34.42±0.42	65.57±1.05

Data are expressed as mean ±SD, n=3.

In the same context, PCSeNPs were used also at concentrations of 0–50 μg/mL and the lowest concentration (3.13 μg/mL) recorded the highest viability of 86.50% ± 0.62% and lower inhibition effect of 13.5% ± 0.22%, while with the highest inhibition of 65.57% ± 1.05% recorded at the highest PCSeNPs (50 μg/mL) (Table 4).

### 3.8 *In vitro* cell cycle analysis of free *Polycladia crinita* extract and PCSeNPs using flow cytometry

**FIGURE 2:**
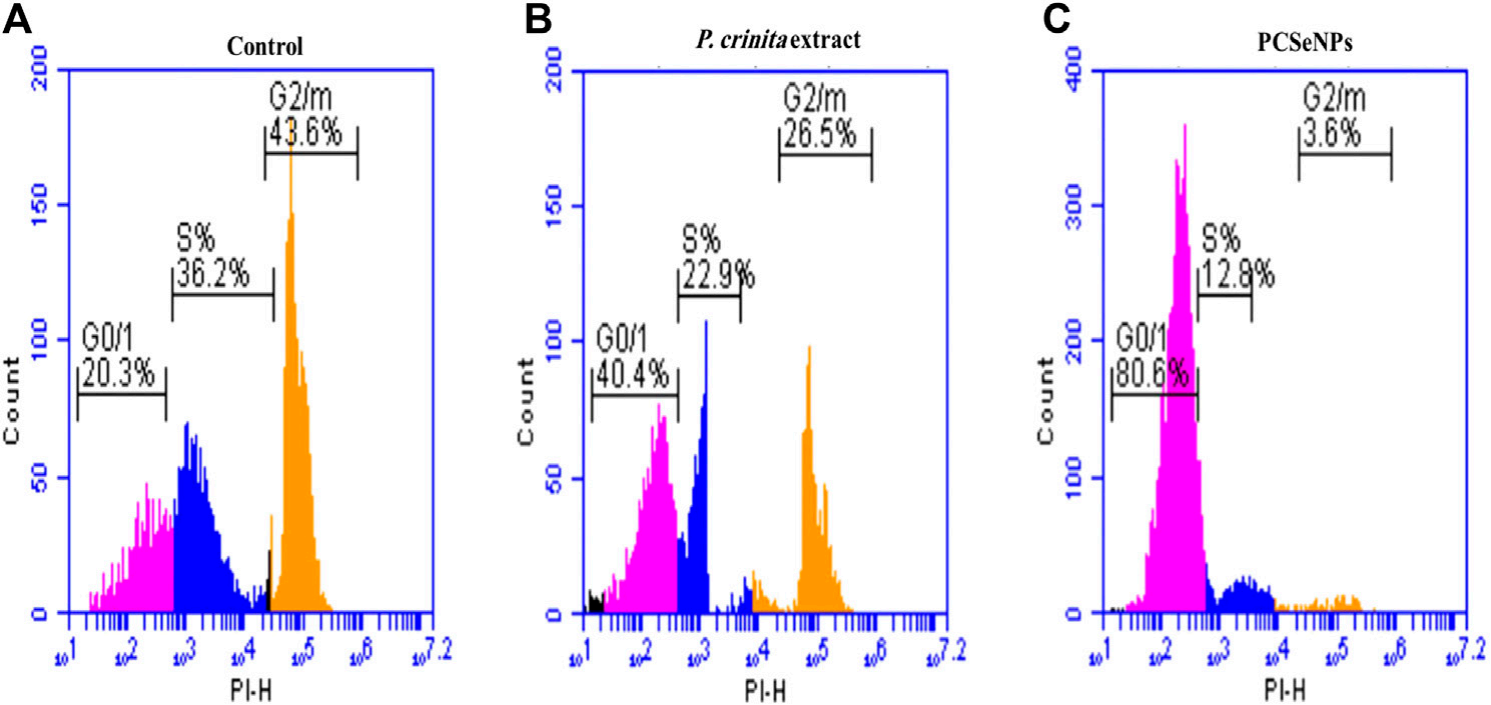
The breast cancer cell line (MDA-MB-231) cell-cycle distribution. Control **(A)**, after treatment with Polycladia crinita extract **(B)**, and PCSeNPs **(C)**, analyzed using flow cytometric analysis on the IC_50_ concentrations detected by MTT assay.

The cell cycle analysis of free *P. crinita* extract and PCSeNPs using flow cytometry is shown in Figure 2 (A, B, and C), in different cell cycle phases (G0/G1, S, and G2/M). Treatment with free *P. crinita* extract indicated G2/M phase cell cycle arrest from 43.6% in untreated cells to 26.5% in the treated cells. In the same context, cells treated with PCSeNPs showed sharp G2/M phase cell cycle arrest from 43.6% in untreated cells to 3.6% in the treated cells.

### 3.9 *Polycladia crinita* SeNPs effect on survival rate and tumor weight

**FIGURE 3:**
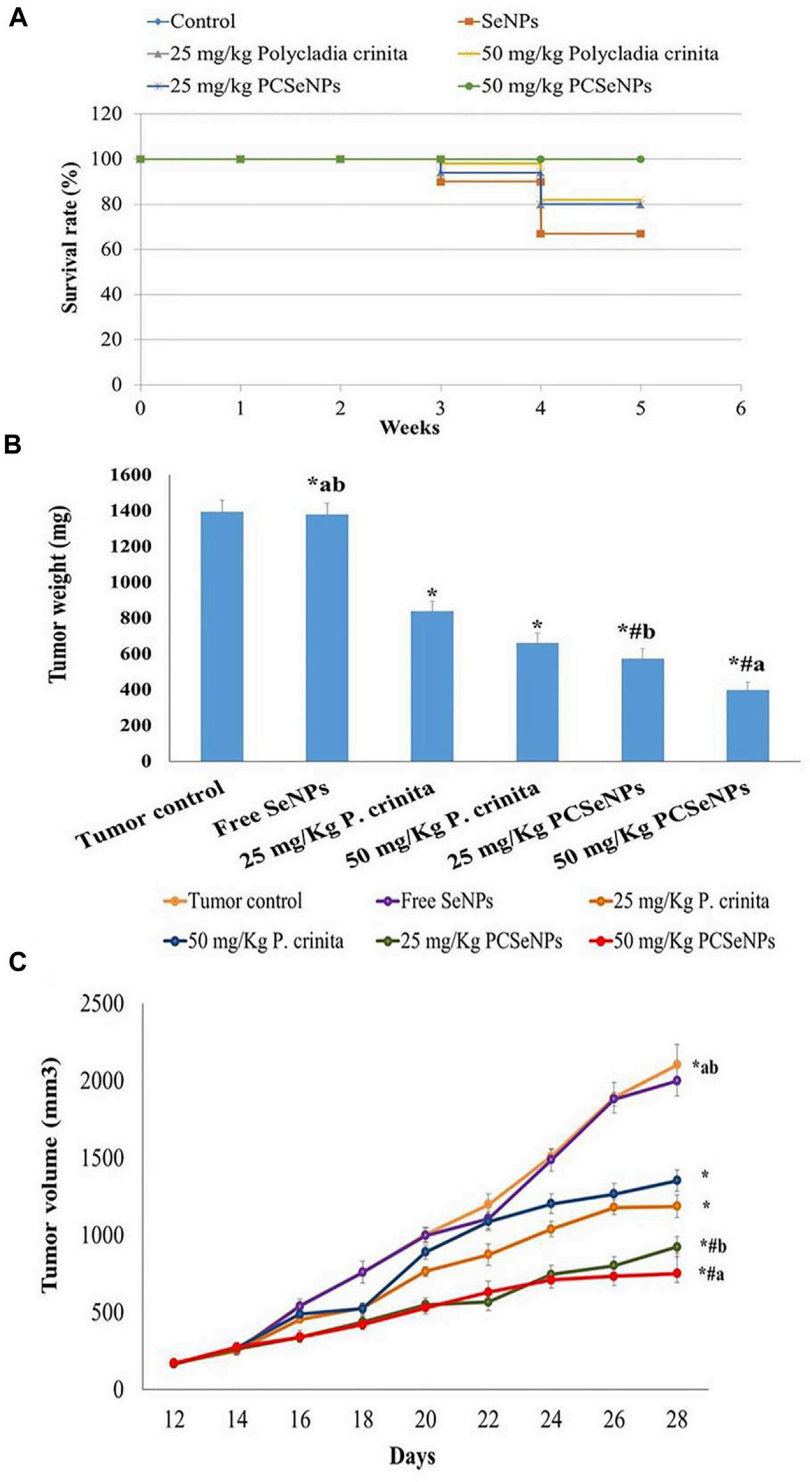
**(A)** The survival rate of mice in each group described by Kaplan-Meier survival plots. The survival rate of each group was calculated according to the formula: survival rate = number of surviving mice/total number of mice during the experiment (28 days) after 1, 2, 3, and 4 weeks. **(B)** Effect of treatments tumor weight. Data are expressed as mean ± SD, n = 6. ^*^means significant *versus* the tumor control group, ^#^means significant *versus* the free SeNPs group, ^a^means significant *versus* 25 mg/kg PCSeNPs group, and ^b^means significant *versus* 50 mg/kg PCSeNPs group. SeNPs: selenium nanoparticles, *Polycladia crinita*: *Polycladia crinita*, and PCSeNPs: *Polycladia crinita* selenium nanoparticles. Each group differed significantly from the others at *p* ≤ 0.05. **(C)** Effect of various treatments on tumor volume. Data are expressed as mean ± SD, n = 6. ^*^means significant *versus* the tumor control group, ^#^means significant *versus* free SeNPs group, ^a^means significant *versus* 25 mg/kg PCSeNPs group*,* and ^b^means significant *versus* 50 mg/kg PCSeNPs group, SeNPs: selenium nanoparticles, *P. crinita*: *Polycladia crinita*, and PCSeNPs: *Polycladia crinita* selenium nanoparticles. Each group differed significantly from the others at *p* ≤ 0.05.

Mice survival rate was tracked during the experiment and reported using the Kaplan-Meier survival curve. Treatments with a high dose of the free extract showed a 22.39% increase in the survival rate, while the SeNPs loaded extract, with a higher dose, exhibited an increase of survival by 49.3%, compared to the tumor control (untreated) group at the end of the experiment (Figure 3A).

Regarding the tumor weight changes between different treated groups, the free *P. crinita* extract (25, 50 mg/kg) decreased, significantly, the tumor weight, compared to a tumor control group (39.7% and 52.6%, respectively). Additionally, SeNPs loaded with *P. crinita* extract revealed a remarkable decrease in tumor weight (58.9% and 71.5%, respectively) compared with a tumor control group. Tumor weights in a group of 50 mg/kg *P. crinita* selenium nanoparticles (PCSeNPs) were significantly reduced (39.9%), relative to the 50 mg/kg *P. crinita* group (Figure 3B).

### 3.10 *Polycladia crinita* SeNPs effect on tumor volume

The tumor volume of the untreated mice showed a gradual increase from day 12 until day 28, with a volume equal to 2,104.3 mm^3^. On the other hand, treating mice with the free *P. crinita* extract (25, 50 mg/kg) significantly decreased the tumor volume (35.6%, and 43.5%, respectively), compared to a tumor control group. Likewise, the SeNPs loaded *P. crinita* extract (25, 50 mg/kg) showed a significant suppression (56.03%, and 64.2%, respectively) compared to the untreated group (Figure 3C).

### 3.11 *Polycladia crinita* SeNPs effect on VEGF, NF-кB, notch 1, and cyclin D1 content

**FIGURE 4:**
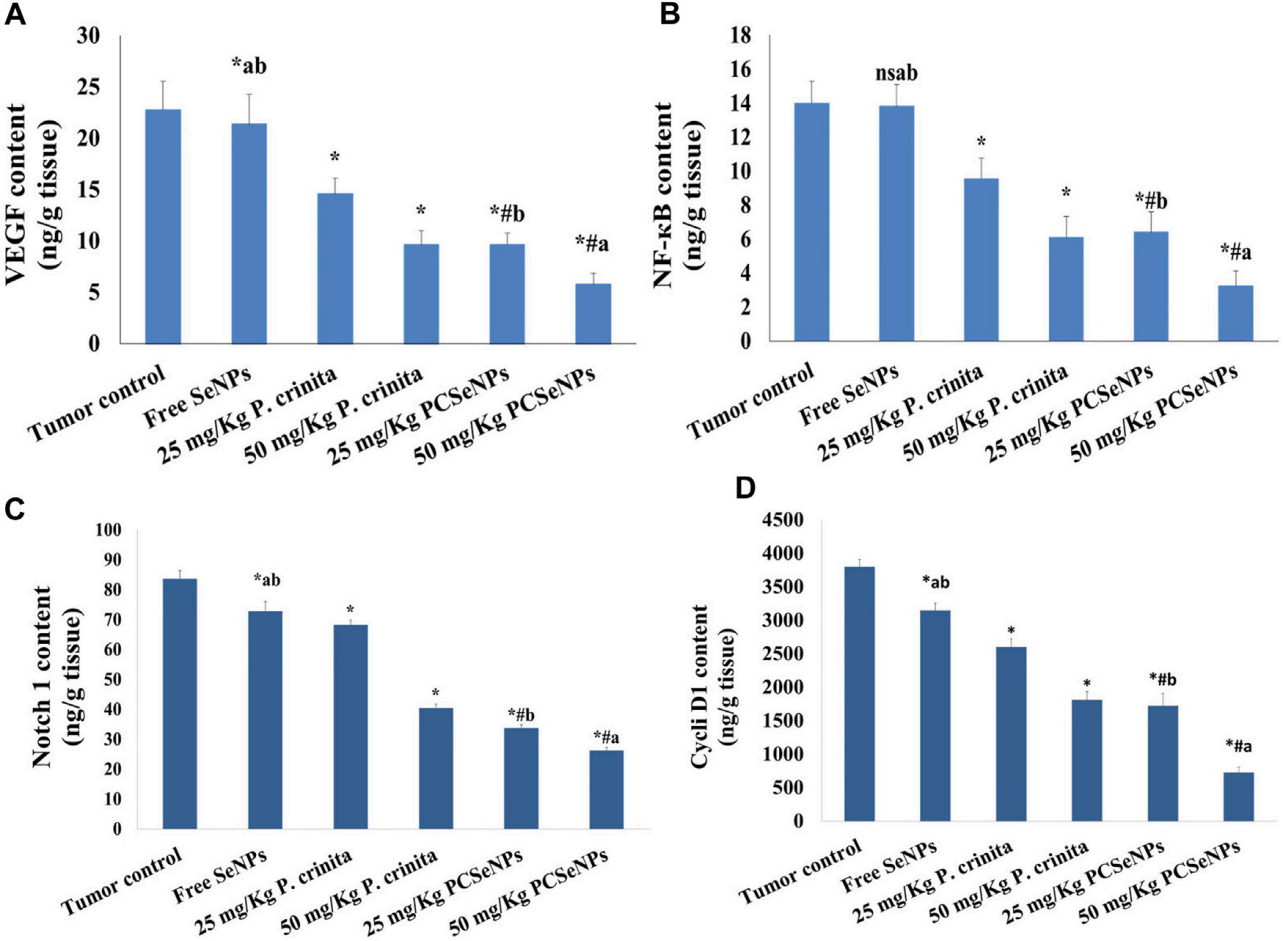
Effect of treatments on VEGF **(A)**, NF-кB **(B)**, Notch 1 **(C)**, and Cyclin D1 **(D)** protein levels using ELISA technique at the end of the experiment (28 days). Data are expressed as mean ± SD, n = 6. ^*^means significant *versus* the tumor control group, ^#^means significant *versus* free SeNPs group, ^a^means significant *versus* 25 mg/kg PCSeNPs group*,* ^b^means significant *versus* 50 mg/kg PCSeNPs group, and ^ns^: means non-significant *versus* control group. SeNPs: selenium nanoparticles, *Polycladia crinita*: *Polycladia crinita*, and PCSeNPs: *Polycladia crinita* selenium nanoparticles. Each group differed significantly from the others at *p* ≤ 0.05.

Figure 4 (A, B, C, and D) shows the effect of different treatments on the protein level of VEGF, NF-кB, Notch 1, and Cyclin D1 in tumor tissue. Groups of free *P. crinita* extract (25, 50 mg/kg) showed a profound decrease in VEGF protein levels (35.9% and 57.7%, respectively), compared to a tumor control group. Furthermore, groups SeNPs loaded with *P. crinita* extract (25, 50 mg/kg) significantly, suppressed VEGF protein levels (57.6% and 74.6%, respectively) relative to a tumor control group (Figure 4A).

Treating mice bearing SEC with free *P. crinita* extract (25, 50 mg/kg) revealed a sustainable decrease in NF-кB protein values (31.8% and 56.5%, respectively), following the tumor control group. Additionally, groups SeNPs loaded *P. crinita* extract (25, 50 mg/kg) revealed a more significant decrease in the NF-кB levels (54.3% and 76.8%), in comparison with untreated SEC-mice (tumor control group) (Figure 4B).

In the same context, Treating mice bearing SEC with free *P. crinita* extract (25, 50 mg/kg) revealed a sustainable decrease in Notch 1 protein values (18.4% and 51.6%, respectively), following the tumor control group. Also, groups SeNPs loaded *P. crinita* extract (25, 50 mg/kg) revealed a more significant decrease in the Notch 1 levels (59.7% and 68.7%), in comparison with untreated SEC-mice (tumor control group) (Figure 4C).

Furthermore, Treating mice bearing SEC with free *P. crinita* extract (25, 50 mg/kg) revealed a sustainable decrease in Cyclin D1 protein values (31.5% and 52.3%, respectively), following the tumor control group. In addition, groups SeNPs loaded *P. crinita* extract (25, 50 mg/kg) revealed a more significant decrease in the Cyclin D1 levels (54.7% and 80.8%), in comparison with untreated SEC-mice (tumor control group) (Figure 4D).

### 3.12 *Polycladia crinita* SeNPs effect on caspase 3, caspase 9, notch 1, cyclin D1, NF-кB, IL-6 and VEGF gene expression

**FIGURE 5:**
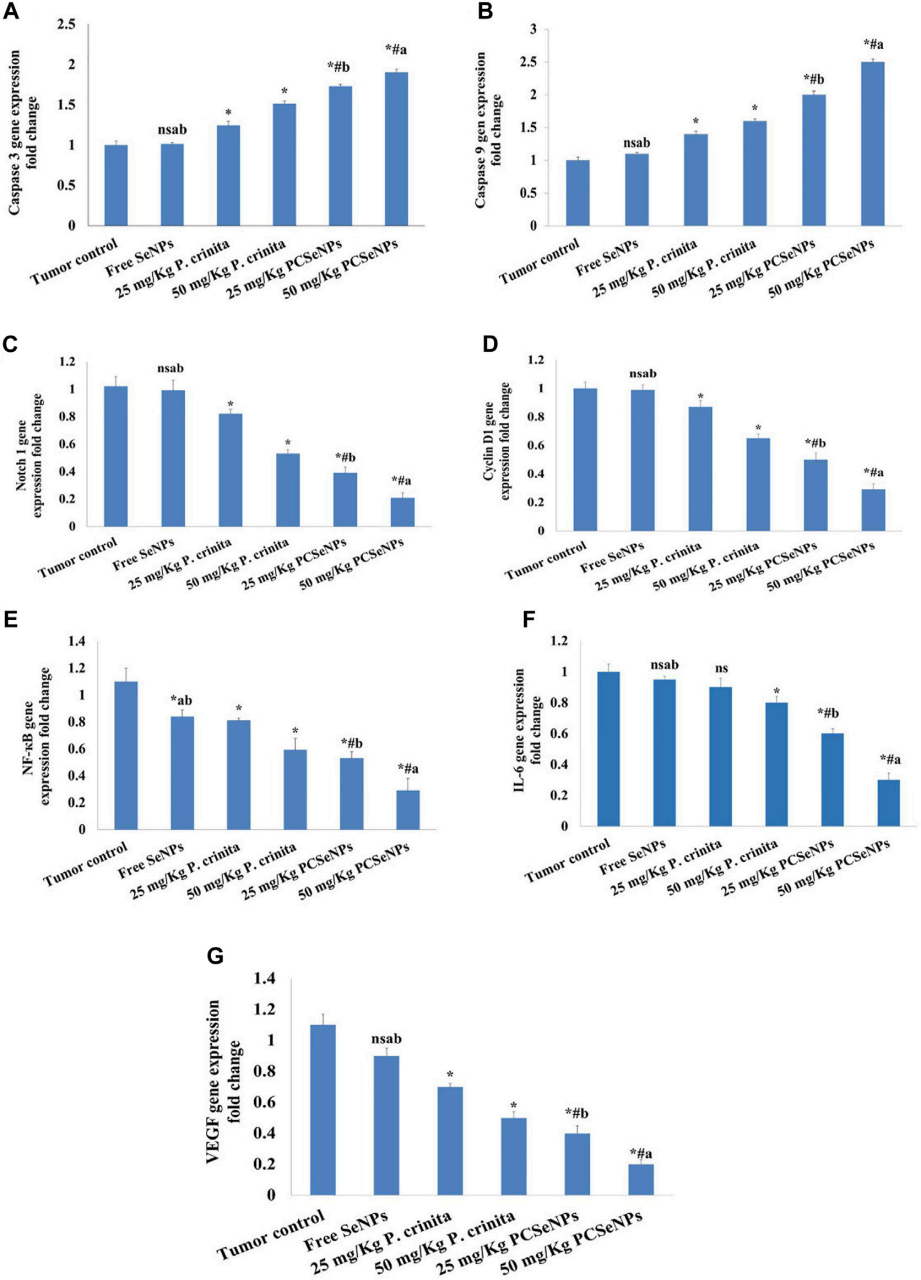
Effect of treatments on Caspase 3 **(A)**, Caspase 9 **(B)**, Notch1 **(C)**, Cyclin D1 **(D)**, NF-кB **(E)**, IL-6 **(F)** and VEGF **(G)** gene expression using the qRT-PCR technique at the end of the experiment (28 days). Data are expressed as mean ± SD, n = 6. ^*^means significant *versus* the tumor control group, ^#^means significant *versus* free SeNPs group, ^a^means significant *versus* 25 mg/kg PCSeNPs group*,* ^b^means significant *versus* 50 mg/kg PCSeNPs group, and ^ns^: means non-significant *versus* a control group. SeNPs: selenium nanoparticles, *Polycladia crinita*: *Polycladia crinita*, and PCSeNPs: *Polycladia crinita* selenium nanoparticles. Each group differed significantly from the others at *p* ≤ 0.05.

Treating mice with *P. crinita* free extract (25, 50 mg/kg), revealed a remarkable enhancement in the caspase 3 gene expression (19.35%, and 33.77%, respectively) related to a tumor control group. Additionally, mice that received the SeNPs loaded with *P. crinita* free extract doses showed a significant increase (73%, and 90%, respectively), in comparison with the tumor control (Figure 5A).

Mice treated with *P. crinita* free extract (25, 50 mg/kg), exposed a significant improvement in the caspase 9 gene expression (40%, and 60%, respectively) related to a tumor control group. Additionally, mice that received the SeNPs loaded with *P. crinita* free extract doses showed a significant rise (100%, and 150%, respectively), in comparison with the tumor control (Figure 5B).

Regarding the gene expression of Notch1, *P. crinita* free extract (25, 50 mg/kg) groups significantly suppressed the change by (19.61%, and 48.04%, respectively), as relative to a tumor control group. Similarly, groups of SeNPs loaded *with P. crinita* extract (25, 50 mg/kg) showed Notch1 expression lessened (56.4% and 72.3%, %, respectively) when compared with a tumor control group (Figure 5C). In the context, cyclin D1 gene expression decreased after treatments with *P. crinita* free extract (25, 50 mg/kg) and SeNPs loaded *P. crinita* extract (25, 50 mg/kg) by (13, 35, 50, and 71% decrease, respectively), compared to a tumor control group (Figure 5D).

Additionally, *P. crinita* free extract (25, 50 mg/kg), showed a significant decrease in the NF-кB gene expression (26.36%, and 41%, respectively) related to a tumor control group. Additionally, mice that received the SeNPs loaded with *P. crinita* free extract doses showed a significant increase (57%, and 81%, respectively), in comparison with the tumor control (Figure 5E).

Likewise, *P. crinita* free extract (25, 50 mg/kg), revealed a notable decrease in the IL-6 gene expression (10%, and 20%, respectively) related to a tumor control group. Additionally, mice that received the SeNPs loaded with *P. crinita* free extract doses showed a significant increase (40%, and 70%, respectively), in comparison with the tumor control (Figure 5F).

Furthermore, Likewise, *P. crinita* free extract (25, 50 mg/kg), exposed a remarkable decrease in the VEGF gene expression (36.4%, and 54.5%, respectively) related to a tumor control group. Additionally, mice that received the SeNPs loaded with *P. crinita* free extract doses showed a significant increase (63.6%, and 81.8%, respectively), in comparison with the tumor control (Figure 5G).

The SeNPs formulations loaded with *P. crinita* free extract showed significantly more effectiveness than similar doses of free extract in all gene expression (caspase 3, caspase 9, Notch 1, Cyclin D1, NF-кB, IL-6, and VEGF).

### 3.13 Histopathological evaluation

**FIGURE 6:**
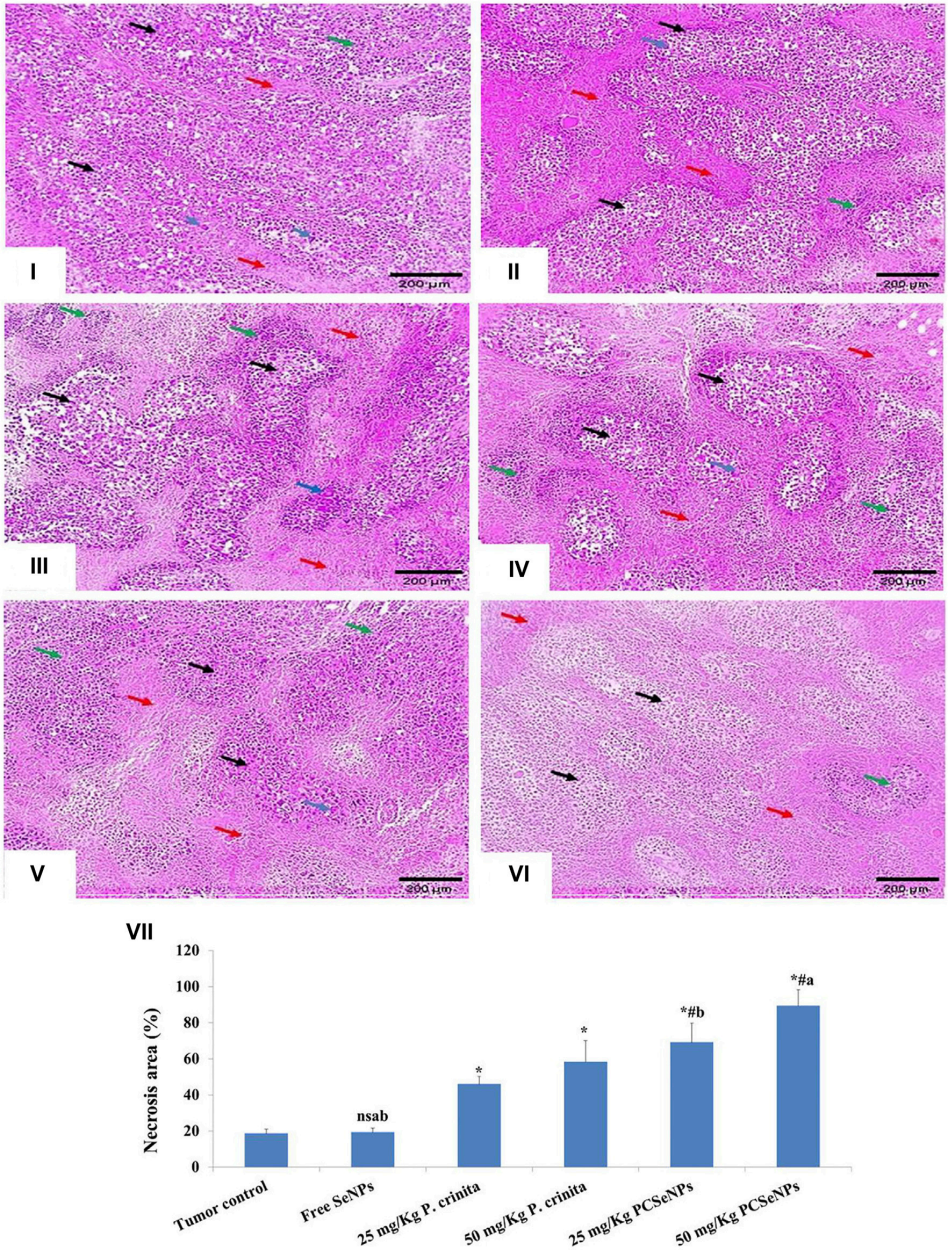
Histopathological findings (100x, scale bar = 200 μm). (i): Tumor control group showed solid sheets of malignant cells (black arrows), many tumor giant cells (blue arrow) with a mild area of necrosis grade 1 (red arrows), and mild lymphocytic infiltrate (green arrow). (ii): Free SeNPs group showed malignant cells (black arrows), many tumor giant cells (blue arrows) surrounded by mild tumor necrosis (red arrows) [necrosis grade 1], and mild lymphocytic infiltrate (green arrows). (iii): 25 mg/kg *Polycladia crinita* group showed malignant cells (black arrows), some tumor giant cells (blue arrows) surrounded by moderate tumor necrosis (red arrows) [necrosis grade 2], and mild lymphocytic infiltrate (green arrows). (iv): 50 mg/kg *Polycladia crinita* group showed malignant cells (black arrows), some tumor giant cells (blue arrows) surrounded by marked tumor necrosis (red arrows) [necrosis grade 3], and moderate lymphocytic infiltrate (green arrows). (v): 25 mg/kg PCSeNPs group showed malignant cells (black arrows), a few tumor giant cells (blue arrows) surrounded by marked tumor necrosis (red arrows) [necrosis grade 3], and moderate lymphocytic infiltrate (green arrows). (vi): 50 mg/kg PCSeNPs group showed shadows of necrotizing malignant and giant cells (black arrows) surrounded by diffuse tumor necrosis (red arrows) [necrosis grade 4] and moderate lymphocytic infiltrate (green arrow). (vii): Necrosis area (%) in tumor sections stained with H&E staining. Quantification of area% was done by ImageJ software; version 1.54 D, Java 1.8.0_354. Data are expressed as mean ± SD, n = 6. ^*^means significant *versus* the tumor control group, ^#^means significant *versus* free SeNPs group, ^a^means significant *versus* 25 mg/kg PCSeNPs group*,* ^b^means significant *versus* 50 mg/kg PCSeNPs group, and ^ns^: means non-significant *versus* control group. SeNPs: selenium nanoparticles, *Polycladia crinita*: *Polycladia crinita*, and PCSeNPs: *Polycladia crinita* selenium nanoparticles. Each group differed significantly from the others at *p* ≤ 0.05.

The untreated mice showed sheets of malignant cells, many tumor giant cells with a mild area of necrosis grade 1, and mild lymphocytic infiltrate. On the other hand, treated groups showed enhancement in the histopathological changes with increased necrotic grades (Figure 6). In the same context, the necrotic area (%) was assessed in H&E and showed induction in all treated groups (Figure 6), especially with a high dose of SeNPs loaded with *P. crinita* free extract.

### 3.14 Immunohistochemical evaluation

**FIGURE 7:**
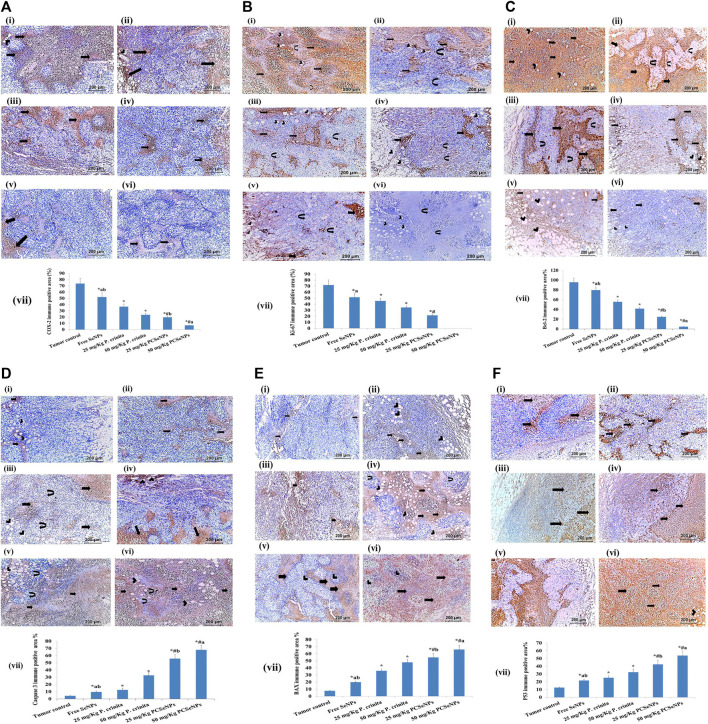
**(A)**. COX-2 immunohistochemical expression (100x, scale bar = 200 μm). (i): Tumor control group showed moderate immune reaction (arrows) all over the tumor and in the vicinity of fat cells (arrowhead) invading the tumor. (ii): Free SeNPs group showed moderate immune reaction (arrows) all over the tumor and in the vicinity of fat cells (arrowhead) invading the tumor. (iii): 25 mg/kg *Polycladia crinita* group showed slightly moderate immune reaction (arrows) all over the tumor. (iv): 50 mg/kg *Polycladia crinita* group showed mild immune reaction (arrows). (v): 25 mg/kg PCSeNPs group showed mild immune reaction (arrows). (vi): 50 mg/kg PCSeNPs group showed very weak immune reaction (arrows) all over the tumor. (vii): COX-2 immunohistochemical positive area (%). Quantification of area% was done by ImageJ software; version 1.54 D, Java 1.8.0_354. Data are expressed as mean ± SD, n = 6. ^*^means significant *versus* the tumor control group, ^#^means significant *versus* free SeNPs group, ^a^means significant *versus* 25 mg/kg PCSeNPs group, and ^b^means significant *versus* 50 mg/kg PCSeNPs group. SeNPs: selenium nanoparticles, *Polycladia crinita*: *Polycladia crinita*, and PCSeNPs: *Polycladia crinita* selenium nanoparticles. Each group differed significantly from the others at *p* ≤ 0.05. **(B)**. Ki-67 immunohistochemical expression (100x, scale bar = 200 μm). (i): Tumor control group showed severely positive immune reaction (arrows), in the vicinity of fat cells (arrowheads) and necrotic muscle fibers (curved arrows). (ii): Free SeNPs group showed moderately positive immune reaction (arrows), in the vicinity of fat cells (arrowheads) and muscle fibers (curved arrows). (iii): 25 mg/kg *P. crinita* group showed moderately positive immune reaction (arrows), in the vicinity of fat cells (arrowheads) and muscle fibers (curved arrows). (iv): 50 mg/kg *P. crinita* group showed mild immune reaction (arrows), in the vicinity of fat cells (arrowheads) and muscle fibers (curved arrows). (v): 25 mg/kg PCSeNPs group showed mild positive immune reaction (arrows), in the vicinity of fat cells (arrowheads). (vi): 50 mg/kg PCSeNPs group showed a negative immune reaction of the tumor muscle fibers (curved arrows) which are invaded by fat cells (arrowheads). (vii): Ki-67 immunohistochemical positive area (%). Quantification of area% was done by ImageJ software; version 1.54 D, Java 1.8.0_354. Data are expressed as mean ± SD, n = 6. ^*^means significant *versus* the tumor control group, ^#^means significant *versus* free SeNPs group, and ^a^means significant *versus* 25 mg/kg PCSeNPs group. SeNPs: selenium nanoparticles, *P. crinita*: *Polycladia crinita*, and PCSeNPs: *Polycladia crinita* selenium nanoparticles. Each group differed significantly from the others at *p* ≤ 0.05. **(C)**. Bcl-2 immunohistochemical expression (100x, scale bar = 200 μm). (i): Tumor control group had severe positive reactions (arrows) and in the vicinity of fat cells (arrowheads). (ii): Free SeNPs group showed strongly positive reaction (arrows) around and in-between necrotic muscle fibers (curved arrows). (iii): 25 mg/kg *P. crinita* group showed a moderately positive reaction (arrows) in the vicinity of necrotic muscle fibers (curved arrows). (vi): 50 mg/kg *P. crinita* group showed moderately positive reaction (arrows) around and in-between fat cells (arrowheads) invading the tumor. (v): 25 mg/kg PCSeNPs group showed mildly positive immune reaction (arrows) around and in-between fat cells (arrowheads) invading the tumor. (vi): 50 mg/kg PCSeNPs group showed weak immune reaction (arrows) around and in-between fat cells (arrowheads) invading the tumor. (vii): Bcl-2 immunohistochemical positive area (%). Quantification of area% was done by ImageJ software; version 1.54 D, Java 1.8.0_354. Data are expressed as mean ± SD, n = 6. ^*^means significant *versus* the tumor control group, ^#^means significant *versus* free SeNPs group, ^a^means significant *versus* 25 mg/kg PCSeNPs group, and ^b^means significant *versus* 50 mg/kg PCSeNPs group. SeNPs: selenium nanoparticles, *P. crinita*: *Polycladia crinita*, and PCSeNPs: *Polycladia crinita* selenium nanoparticles. Each group differed significantly from the others at *p* ≤ 0.05. **(D)**. Caspase 3 immunohistochemical expression (100x, scale bar = 200 μm). (i): Tumor control group showed weak positive reaction (arrows) in the vicinity of fat cells (arrowheads) all over the tumor and in-between and fat cells (arrowheads) invading the tumor. (ii): Free SeNPs group showed mild positive reaction (arrows) all over the tumor. (iii): 25 mg/kg *P. crinita* group showed mild positive reaction (arrows) all over the tumor, in-between muscle fibers (curved arrows) and fat cells (arrowheads) invading the tumor. (vi): 50 mg/kg *P. crinita* group showed strong moderate positive reaction (arrows) all over the tumor. (v): 25 mg/kg PCSeNPs group strong positive reaction (arrows) all over the tumor, in-between muscle fibers (curved arrows) and fat cells (arrowheads) invading the tumor. (vi): 50 mg/kg PCSeNPs group showed strong positive reaction (arrows) all over the tumor, in-between muscle fibers (curved arrows) and fat cells (arrowheads) invading the tumor. (vii): Caspase 3 immunohistochemical positive area. Quantification of area% was done by ImageJ software; version 1.54 D, Java 1.8.0_354. Data are expressed as mean ± SD, n = 6. ^*^means significant *versus* the tumor control group, ^#^means significant *versus* free SeNPs group, ^a^means significant *versus* 25 mg/kg PCSeNPs group, and ^b^means significant *versus* 50 mg/kg PCSeNPs group. SeNPs: selenium nanoparticles, *P. crinita*: *Polycladia crinita*, and PCSeNPs: *Polycladia crinita* selenium nanoparticles. Each group differed significantly from the others at *p* ≤ 0.05. **(E)**. BAX immunohistochemical expression (100x, scale bar = 200 μm). (i): Tumor control group showed weak mild immune reaction (arrows) all over the tumor. (ii): Free SeNPs group showed weak mild immune reaction (arrows) all over the tumor and in the vicinity of fat cells (arrowheads). (iii): 25 mg/kg *P. crinita* group showed mild positive immune reaction (arrows) all over the tumor and in-between muscle fibers (arrowheads). (vi): 50 mg/kg *P. crinita* group showed strong moderate immune reaction (arrows) all over the tumor, in the vicinity of fat cells (arrowheads) and in-between muscle fibers (curved arrows). (v): 25 mg/kg PCSeNPs group showed moderate immune reaction (arrows) all over the tumor and in the vicinity of muscle fibers (arrowheads). (vi): 50 mg/kg PCSeNPs group showed severe immune reaction (arrows) all over the tumor. (vii): BAX immunohistochemical positive area. Quantification of area% was done by ImageJ software; version 1.54 D, Java 1.8.0_354. Data are expressed as mean ± SD, n = 6. ^*^means significant *versus* the tumor control group, ^#^means significant *versus* free SeNPs group, ^a^means significant *versus* 25 mg/kg PCSeNPs group, and ^b^means significant *versus* 50 mg/kg PCSeNPs group. SeNPs: selenium nanoparticles, *P. crinita*: *Polycladia crinita*, and PCSeNPs: *Polycladia crinita* selenium nanoparticles. Each group differed significantly from the others at *p* ≤ 0.05. **(F)**. P53 immunohistochemical expression (100x, scale bar = 200 μm). (i): Tumor control group showed mild positive reactions (arrows) all over the tumor. (ii): Free SeNPs group showed mild positive reaction (arrows) all over the tumor. (iii): 25 mg/kg *P. crinita* group showed moderate positive reaction (arrows) all over the field and in the vicinity of fat cells (arrowhead). (iv): 50 mg/kg *P. crinita* group showed moderate positive reaction (arrows) all over the tumor. (v): 25 mg/kg PCSeNPs group showed strong moderate positive reaction (arrows) all over the tumor. (vi): 50 mg/kg PCSeNPs group showed strong positive reactions (arrows) all over the tumor and in the vicinity of fat cells (arrowheads). (vii): P53 immunohistochemical positive area (%). Quantification of area% was done by ImageJ software; version 1.54 D, Java 1.8.0_354. Data are expressed as mean ± SD, n = 6. ^*^means significant *versus* the tumor control group, ^#^means significant *versus* free SeNPs group, ^a^means significant *versus* 25 mg/kg PCSeNPs group, and ^b^means significant *versus* 50 mg/kg PCSeNPs group. SeNPs: selenium nanoparticles, *P. crinita*: *Polycladia crinita*, and PCSeNPs: *Polycladia crinita* selenium nanoparticles. Each group differed significantly from the others at *p* ≤ 0.05.

Immunohistochemical staining for the inflammation marker (COX-2), Proliferation marker (Ki-67), and antiapoptotic marker (Bcl-2) showed intense staining in the tumor control group. On the other hand, different treatments showed a significant reduction in immunoreactivity as compared to the tumor control group (Figures 7A–C). Otherwise, apoptotic markers (caspase 3, BAX, and P53) showed a significant decline in immunoreactivity in the tumor control group, while, showed a significant strong in immunoreactivity appeared with different treatments as compared to the tumor control group (Figures 7D–F).

## 4 Discussion

Since *Polycladia crinita* is recognized as a treasure of promising bioactive compounds selenium nanoparticles show low toxicity and high biocompatibility (Shaheen and Fouda, 2018; Soliman et al., 2021). Additionally, the promising antioxidant and cytotoxic activities along with the unique physicochemical properties and kinetic stability of nano-emulsion compared to its bulk materials have directed us to investigate the possibility of using nano-emulsion as an alternative, used with low or no toxic side effects, anticancer agent for the traditional chemotherapeutics (Menon et al., 2018). The current study was conducted to evaluate the potential *in vitro* and *in vivo* the potential anti-cancer effect of *polycladia crinita*, as a free extract or SeNPs-loaded one. *Polycladia crinita* extract revealed the existence of many bioactive components such as 4-octadecenoic acid-methyl ester, tetradecanoic acid, and n-Hexadecenoic acid using GC-MS analysis. These compounds were mainly characterized as fatty acids (FA) (Casula et al., 2013). Fatty acids have many beneficial effects on human health (Kyle et al., 1999; Serini et al., 2009; Wall et al., 2010; Harris et al., 2013; Bi et al., 2019). The biosynthesized PCSeNPs were characterized by various analytical procedures, like SEM, TEM, EDX, and XRD.

The free *P. crinita* extract and PCSeNPs have antitumor activity and sharp G2/M phase cell cycle arrest against the breast cancer cell line (MDA-MB-231) with special remarkably to PCSeNPs. However, there is still more to learn about the SeNPs anticancer mechanism. Nonetheless, several writers highlight the various effects of selenium nanoparticles (SeNPs) on cancer cells, including their penetration, inhibition of certain cancer-induced enzymes like EGFR, regulation of ROS production, induction of autophagy, and stimulation of cancer cell apoptosis (Gao et al., 2020; Makhlof et al., 2022).

Here, injecting EAC cells in mice created solid tumors that increased in volume within the subsequent 2 weeks. The untreated mice developed a solid tumor that gradually increased in size and weight till the end of the experiment, which was consistent with previous research (Roelofs et al., 2014). Also, these mice showed histopathological findings revealing solid sheets of malignant cells and many tumor giant cells (Fosslien, 2000). On the other side, upon receiving *Polycladia crinita*, either free extract or SeNPs loaded one, distinctly suppressed tumor volume and weight parallel with enhancing the animals’ survival rate. Similarly, an earlier study speculated the inhibitory effect of *Polycladia myrica* against cancer cells (Wang B. et al., 2012). Supporting the aforementioned findings, the histopathological examination showed shadows of necrotizing cells.

In the current study, the positive control group showed a highly expressed Ki-67 protein, an effect that was counteracted by Polycladia extract either free or nanoformulated. Our data were in line with a previously conducted study, where Ki-67 expression was profoundly inhibited after oleic acid treatment of colon cancer (Deng et al., 2023). Given the presence of oleic acid as a component of Polycladia Crinita, herein, this may give a possible explanation for the effect of *Polycladia* on Ki-67 tissue expression. Ki-67 is frequently utilized as a proliferation marker for breast cancer since it is significantly connected with tumor cell proliferation and expansion. Also, malignant tissues with poorly differentiated tumor cells have much increased Ki-67 expression when compared to normal tissue (Wang et al., 2015; Gheda et al., 2021).

Vascular endothelial growth factor (VEGF) is a master regulator of angiogenesis that is required for the viability and ruthless growth of solid tumors like breast cancer (Aldubayan et al., 2019). In breast cancer cells, there is an imbalance between angiogenesis and apoptosis (Gardouh et al., 2020). Herein, treating SEC-bearing mice with the algae extract profoundly suppressed VEGF expression, and hence angiogenesis. Notably, the SeNPs loaded formulation showed the upper hand, in decreasing VEGF expression, over the same dose of free extract. The possible explanation here is the presence of fenretinide in *Polycladia* characterization, which showed in earlier studies an inhibitory effect on VEGF (Sogno et al., 2010).

Nuclear factor kappa B (NF-кB) is a transcription factor, which upon activation by ROS; regulates different processes such as pro-inflammatory, proliferation, apoptosis, and survival by mediating the expression of several molecules including cytokines (IL-6, COX-2,. etc.) (Serini et al., 2009). Thus, it has been introduced as a main target in controlling tumor growth (Co et al., 2019). In the current study, free and SeNPs loaded extract of *Polycladia crinita* profoundly decreased NF-ҡB expression, which might be explained by the ability of the algae to inhibit oxidative stress. These findings were confirmed by (Shaheen and Fouda, 2018), who stated the antioxidative effect of *Polycladia myrica* in the liver. Interestingly*,* the SeNPs loaded dose proved a preferable effect compared with the counterpart dose of the free extract (Menon et al., 2018), similarly (Makhlof et al., 2022), stated that nanoformulation was superior to the free ones.

Cyclooxygenases (COXs) are crucial regulators of cell proliferation, through transforming free arachidonic acid into prostaglandin-H2 (Mukherjee et al., 2001), a precursor of other prostaglandins, which participate in immunological response, inflammation, apoptosis, and angiogenesis, all of which aid in the onset or development of cancer (Poinern, 2014). A key player in the emergence of epithelial cell carcinoma and metastasis is the inducible enzyme COX-2 (Salem and Fouda, 2021). COX-2-positive tumors are more likely to have a poorer prognosis and to be more aggressive, so many types of cancer may be controlled by selectively inhibiting COX-2 expression (Salem and Fouda, 2021). The synthesis of COX-2 is significantly regulated by the abundant and inducible cell transcription factor NF-ҡB (Vikneshan et al., 2020). Herein, COX2 expression was increased in the untreated group, while dosing mice with free or nano-loaded extracts exhibited a notable decrease in COX2 protein. Our earlier study was in agreement, where Polycladia imposed a profound suppression of COX2 expression in Carrageenan-induced paw edema (Siddiqui et al., 2011).

Apoptosis is defined as programmed cell death that, by removing unstable cells, maintains tissue stability. Caspase 3, caspase 9, P53, and BAX are the main players in the context of activating the apoptotic cascades and Bcl2 is an antiapoptotic mediator. In cancer, apoptosis is downregulated, and cell growth is increased, which drives both tumor proliferation and growth (Zhong et al., 2016). The present results confirmed this, as presented by the continuing increase of the tumor volume in the untreated group. Additionally, this group exhibited a weak gene expression of caspase 3, BAX, and p53 and strong Bcl2 expression. Treating mice with *P. crinita*-free extract and PCSeNPs proved its anti-proliferative action by significantly enhancing BAX, p53, and Caspase3 gene expression along with decreasing Bcl2 immunostaining expression. Research that was in agreement with ours regarding the effect of brown algae on cancer p53 expression, a study showed that Polycladia Myrica has significantly lowered p53 expression in MCF7 breast cancer cells (Fahmy et al., 2023).

It was discovered that the expression of p53 and Ki-67 are associated with breast cancer (Karagiannis et al., 2023). It is yet unclear how p53 may impact the expression of the Ki-67 gene. Given that the Ki-67 promoter has three Sp1-binding sites and p53 reduces the transcription of genes at promoters that include Sp1-binding sites (Lansky and Newman, 2007; Johanningsmeier and Harris, 2011). It is most probable that p53 decreases K-i67 promoter activity through Sp1-and p53-dependent pathways. There are thought to be at least two processes that control transcription. One is that the transcriptional suppression of the Ki67 promoter is impacted by the p53-binding motifs. The second involves a potential p53-Sp1 interaction at Sp1-binding sites on the Ki67 promoter.

In the present work, the tumor tissue from mice groups that received treatments manifested a significant suppression in Notch 1 gene expression and a significant decrease in NF-кB levels, an effect that was supported by previously mentioned literature, where Notch acts a vital player in multiple cellular processes including apoptosis, proliferation, and angiogenesis. In breast cancer, the Notch 1 pathway is upregulated and highly correlated with aggressiveness (Didžiapetrienė et al., 2020c). Moreover, Notch activation boosts NF-КB activity, and hence cell proliferation is improved. Remarkably, VEGF and Notch1 dynamically interact at the cellular level to control the tumor’s ability to grow and regenerate, limitlessly (Zhong et al., 2016).

The mentioned interplay between Notch1, NF-кB, and VEGF strongly presents them as distinguished targets in constricting cancer cell overgrowth and explains, logically, the concurrent increase or decrease (as in *Polycladia Crinita* treated groups) of these three mediators in the present study. It is worth mentioning that, to our best knowledge, this study is the first to investigate the *Polycladia* effect on notch1 expression in breast cancer.

Cyclin D1 is a well-known protooncogen whose upregulation means more progressive cancer. It acts as a cell cycle regulatory protein, which is considered one of the downstream targets of the Notch pathway in many cancers such as lung and breast ones. It is worth mentioning that NF-КB is a main mediator of cyclin D1 activity (Johanningsmeier and Harris, 2011; Giulitti et al., 2021). The current study revealed that the untreated mice showed highly expressed cyclin D while treatment with algae extracts profoundly inhibited cyclin D1 expression. A possible explanation could be the appearance of oleic acid, in our characterization, which was proven earlier as a defeater of hepatocellular carcinoma progression through mediating cyclin D1 expression (Giulitti et al., 2021). To our best knowledge, this study is the first to study the effect of *Polycladia crinita* on breast cancer growth and the related drooping of the underlying mediators VEGF, Notch 1, NF-кB, IL-6, Cyclin D1, Caspase 9 and Caspase 3.

## 5 Conclusion

To the best of our knowledge, this study is the first to study the effect of *Polycladia crinita* on breast cancer growth and the related drooping of the underlying mediators VEGF, Notch 1, NF-кB, IL-6, cyclin D1, caspase 9 and caspase 3. *Polycladia crinita* extract, either free or SeNPs, exhibited an antitumoral effect against breast cancer cells of SEC mice, with the nano-formulation having the advantage of exerting a more significant impact above the free extract rival. The *Polycladia crinita* extract mediated its promising anticancerous action by enhancing apoptosis and mitigating inflammation via VEGF, Notch 1, NF-кB, IL-6, Cyclin D1, Caspase 9 and Caspase 3 expression/level, which manifested in promoting the total survival rate and the tumor volume decrease.

## Data Availability

The original contributions presented in the study are included in the article, further inquiries can be directed to the corresponding authors.
